# Genetic polymorphisms in DNA base excision repair gene *XRCC1 *and the risk of squamous cell carcinoma of the head and neck

**DOI:** 10.1186/1756-9966-28-37

**Published:** 2009-03-13

**Authors:** Michal Kowalski, Karolina Przybylowska, Pawel Rusin, Jurek Olszewski, Alina Morawiec-Sztandera, Anna Bielecka-Kowalska, Wioletta Pietruszewska, Wojciech Mlynarski, Janusz Szemraj, Ireneusz Majsterek

**Affiliations:** 1 Department of Chronopharmacology, Medical University of Lodz, Lodz, Poland; 2 Department of Molecular Genetics, University of Lodz, Lodz, Poland; 3 Department of Otolaryngology and Oncology, Medical University of Lodz, Lodz, Poland; 4 Department of Head and Neck Cancer, Medical University of Lodz, Lodz, Poland; 5 Department of Otolaryngology, Medical University of Lodz, Lodz, Poland; 6 Department of Pediatrics, Medical University of Lodz, Lodz, Poland; 7 Department of Medical Biochemistry, Medical University of Lodz, Lodz, Poland

## Abstract

**Background:**

The genes of base excision repair (BER) pathway have been extensively studied in the association with various human cancers. We performed a case-control study to test the association between two common single nucleotide polymorphisms (SNPs) of *XRCC1 *gene with human head and neck squamous cell carcinoma (HNSCC).

**Methods:**

The genotype analysis of Arg194Trp and Arg399Gln gene polymorphisms for 92 HNSCC patients and 124 controls of cancer free subjects, in Polish population were performed using the PCR-based restriction fragment length polymorphism (PCR-RFLP) with endonuclease *Msp*I.

**Results:**

No altered risk has been found individually for these SNPs, however haplotypes analysis showed high association with head and neck cancer. The highest frequency, according to wild-type of Arg194Arg and Arg399Arg genotypes, was identified for Arg194Trp-Arg399Arg haplotype (OR, 2.96; 95% CI, 1.01–8.80).

**Conclusion:**

Finally, we identified the combined Arg194Trp-Arg399Arg genotype of base excision repair gene *XRCC1 *that was associated with HNSCC and may have an impact on identification of a high-risk cancer population.

## Background

Genome integrity is maintained by an intricate network of DNA repair proteins [1,2]. Organisms have developed several DNA repair pathways as well as DNA damage checkpoints. Although each pathway is addressed individually, the cross talk exists between repair pathways, and there are instances in which a DNA-repair protein is involved in more than one pathway. Single nucleotide polymorphisms (SNPs) in DNA repair genes may be associated with differences in the repair efficiency of DNA damage and may influence an individual's risk of cancer. Establishing this connection, however, has been a challenge due to the complexity of interactions that affect the repair pathways [3,4]. Increasing evidence links environmental exposures, subtle modification in DNA repair efficiency, and cancer risk [5].

The genes belonging to base excision repair (BER) pathway, such as X-ray Repair Cross Complementing Group 1 (*XRCC1*) have been extensively studied in the association with various human cancer [6-14]. Two major SNPs of the *XRCC1 *gene have been identified at codon 194 (C > T substitution at position 26304, exon 6, Arg to Trp) and 399 (G > A substitution at position 28152, exon 10, Arg to Gln). The *XRCC1 *Arg399Gln polymorphism is located in the area coding for a PARP binding site. PARP is a zinc-fnger containing enzyme that detects DNA strand breaks [15]. Carriers of the *XRCC*1 399 Gln variant allele have been shown to have higher levels of DNA adducts [16] and to be at greater risk for ionizing radiation sensitivity [17] and tobacco correlated DNA damage [18-20].

The XRCC1 protein plays an important role in the maintenance of genomic stability through the both base excision and single-strand break repair by acting as a scaffold for other DNA repair proteins, such as DNA glycosylases, polymerase beta [21] and ligase III [22]. XRCC1 participates in the first step of BER by interacting with the numerous of human DNA glycosylases including hOGG1, MPG, hNTH1 and NEIL1 [23,24]. It was found that XRCC1, through its NTD and BRCT1 domains, has affinity to form a covalent complex via Schiff base with AP sites. It was also reported that XRCC1 affinity was higher when the DNA carried an AP-lyase- or APE1-incised AP site [25]. This results in an acceleration of the overall repair process of abasic site, which can be used as a substrate by DNA polymerase beta. Thus, this suggests mechanism by which XRCC1, through its multiple protein-protein interactions plays essential role in the resealing of the repaired DNA strand.

Head and neck squamous cell carcinoma (HNSCC) comprise about 6% of all malignant neoplasm. Overall survival is low especially in developing countries and the major risk factors of HNSCC became smoking or alcohol consumption [26]. Although the functional significance of XRCC1 polymorphism has not yet been fully elucidated, due to smoking and alcohol consumption attitude it may increase risk of head and neck cancer occurrence [27]. To test this hypothesis, we performed a hospital based case-control study using a polymerase chain reaction-restriction fragment length polymorphism (PCR-RFLP) assay to genotype two polymorphisms of DNA repair gene *XRCC1 *Arg194Trp and Arg399Gln in relation to head and neck cancer susceptibility.

## Methods

### Patients

Blood samples were obtained from 92 patients (50 men and 42 women, mean age 48.7 ± 11.13) with squamous carcinoma of head and neck. Control samples consisted of age matched 124 cancer-free blood donors (63 men and 61 women, mean age 44.47 ± 19.24). Despite of 4 years younger controls then patients, there were not statistical differences in age of analyzed groups (P = 0.169). Prior to examination, the patients and control subjects, did not receive medicaments like antibiotics or steroids. Patients enrolled to the examination were analyzed according to cancer staging system of the TNM Classification of Malignant Tumours that describes the extent of cancer in a patient's body: T describes the size of the tumor and whether it has invaded nearby tissue, N describes regional lymph nodes that are involved and M describes distant metastasis (spread of cancer from one body part to another). Within the control group selected subjects (52 cases) were classified as smokers for at least 10 years, up to 10 cigarettes per day. The smoking attitude of head and neck cancer group was also analyzed for non-smoking patients, patients smoking 10 cigarettes per day for ten years, patients smoking 20 cigarettes per day for twenty years and patients smoking 20 cigarettes per day for thirty years. All patients and controls subjects were recruited from three medical units of Head and Neck Neoplasm Surgery Departments, Medical University of Lodz, Poland. All subjects included into the study were unrelated Caucasians and inhibited Lodz district, Poland. The study was approved by the Local Ethic Committee and written consent was obtained from each patient or healthy blood donor before enrolling into the study.

### Genotype determination

Genomic DNA was isolated from blood cells using Phenol-Chloroform extraction method. Genotypic analysis of the *XRCC1 *399 G > A polymorphism was determined by the PCR-based restriction fragment length polymorphism (PCR-RFLP) method, as described in detail earlier [28]. Briefly, PCR primers for the *XRCC1 *codon 194 (forward 5'-GCCCCGTCCCAGGTA-3' and reverse 5'-AGCCCCAAGACCCTTTCATC-3') were used to generate a 292 bp product containing the polymorphic sites. PCR primers for the *XRCC1 *codon 399 (forward 5'-TTGTGCTTTCTCTGTGTCCA-3' and reverse 5'-TCCTCCAGCCTTTTCTGATA-3') were used to generate a 615 bp product containing the polymorphic sites. The PCR was carried out in a MJ Research, INC thermal cycler, model PTC-100 (Waltham, MA, USA). The PCR reactions were carried out in a 20 μl volume of 20 pmol of each primer, 0.2 mM each dNTP and 1 μl buffer and 1 U of *Taq *polymerase, with a denaturation of 94°C for 5 min, followed by 30 cycles of 30 s at 61°C and 45 s at 72°C and finally 7 min at 72°C. Following amplification, PCR products were digested using 10 U of restriction enzyme *Msp*I (New England BioLabs, Beverly, MA, USA) for 16 h at 37°C, and electrophoresed on a 3% agarose gel. The wild type Arg allele for codon 194 is determined by the presence of a band at 292 bp, while the mutant Trp allele is determined by the presence of a band at 313 bp (indicative of the absence of the *Msp*I cutting site). In addition to these bands, a 174 bp band, resulting from an additional invariant cutting site for *Msp*I in the 491 bp amplified fragment (codon 194) is always present and serves as internal control for complete *Msp*I digestion. The wild type Arg allele for codon 399 is determined by the presence of two bands at 374 and 221 bp, while the mutant Gln allele is determined by the presence of the uncut 615 bp band (indicative of the absence of the *Msp*I cutting site).

### Data analysis

The allelic frequencies were estimated by gene counting and genotypes were scored. The χ^2 ^test was used to compare the observed numbers of genotypes with those expected for a population in the Hardy-Weinberg equilibrium and to test the significance of the differences of observed alleles and genotypes between groups. The odds ratios (ORs) and 95% confidence intervals (CIs) were calculated by using a logistic regression model. The t-test (for normal distribution) or Manne-Whitney test (for non-normal distribution) was used to compare each parameter between two groups (i.e. sex and age). An analysis of variance test was used to identify parameters that would make significant differences between more than two groups; Scheffe's test was then used to assess the significance of difference in each identified parameter between any two groups. STATISTICA 6.0 software (Statsoft, Tulsa, OK, USA) was used to perform analyses.

## Results and discussion

In this work we investigated two common single nucleotide polymorphisms of *XRCC1 *gene Arg194Trp and Arg399Gln and their association with human head and neck squamous cell carcinoma. The genotype analysis of these two SNPs of *XRCC1 *gene, for 92 HNSCC patients and 124 controls of cancer free subjects, in Polish population were performed using PCR-RFLP method. The polymorphisms chosen for this study have been shown to have functional significance and may be responsible for a low DNA repair capacity phenotype characteristic of cancer patients including head and neck squamous carcinomas [29-32]. The characteristic of HNSCC patens group according to age, sex, tumor stage and smoking status data was displayed in table 1.

**Table 1:** The characteristic of patients group with squamous cell carcinoma of the head and neck (HNSCC).

**Patients**	**Sex**		**Tumor stage** **(TNM)**										**Smoking status** **(cigarettes per day)**			
No.	men	women	T				N				M		non	10cc*	20cc*	20cc***
							
			1	2	3	4	0	1	2	3	0	1				

92	83	9	15	17	31	29	62	15	10	5	92	0	26	9	33	24

T (1–4) – size or direct extent of the primary tumor; N (0–3) – degree of spread to regional lymph nodes; M (0/1) – presence of metastasis; non – not smoking; * – smoking for ten years; ** – smoking for twenty years; *** – smoking for thirty years.

Genome integrity is maintained by an intricate network of DNA repair proteins [33,34]. Organisms have developed several DNA-repair pathways as well as DNA-damage checkpoints. Defects in this complex machinery are associated with genotoxic susceptibility and familial predispositions to cancer [35]. Increasing evidence links environmental exposures, subtle modification in DNA repair efficiency, and cancer risk [36]. XRCC1 participates in DNA single strand break and base excision repair to protect genome stability in mammalian cells. One of the common polymorphisms of *XRCC1 *the Arg399Gln is located in the BRCT1 domain responsible for interacting with other repair components of BER. It was reported that Arg→Gln substitution produces significant conformational changes at BRCT1 domain that may be critical for DNA repair protein-protein interactions [37], thus absence or impairment repair may cause genome instability and cancer occurrence. It is also important to integrate DNA-repair process with DNA-damage checkpoints and cell survival, to evaluate the role of DNA repair at both cellular and organismic levels. Therefore, protective effects of XRCC1 polymorphisms in cancer may also be observed by the enhanced efficiency of apoptosis at a cellular level as a result of diminished DNA repair capacity secondary to the genetic polymorphisms [38,39].

In our study, neither of these SNPs was found to individually contribute to head and neck cancer risk. There were no differences between the distribution of the genotypes or alleles frequences in patients and controls. However, we found statistically non-significant increase of Arg194Trp genotype frequency (OR, 1.37; 95% CI, 0.70–2.68) and Trp^194 ^allele (OR, 1.32; 95% CI, 0.70–2.49) according to wild-type of Arg194Arg reference genotype and Arg^194 ^allele frequency (table 2). Non-statistical increase of Arg399Gln (OR, 1.10; 95% CI, 0.61–1.97) according to reference genotype of Arg399Arg was also found. (table 3). While, no altered risk has been found individually for the *XRCC1 *Arg194Trp or Arg399Gln polymorphisms, the halophyte analysis according to wild-type of Arg194Arg-Arg399Arg showed high association with head and neck cancer (table 4). The findings indicated that a statistically non-significantly increased risk of HNSCC was associated with the combined Arg194Arg-Arg399Gln genotype (OR, 1.33; 95% CI, 0.70–2.56). The higher risk of head and neck cancer occurrence was associated with the combined Arg194Trp-Arg399Arg genotype (OR, 2.96; 95% CI, 1.01–8.80) but no altered risk was associated with others haplotypes. For Tyr165Tyr genotype we also observed positive correlation with cancer progression assessed by tumor size (OR 4.56; 95% CI 1.60–12.95).

**Table 2:** Distribution of genotypes and frequency of alleles of the Arg/Trp 194 (C/T 26304 exon 6) polymorphism of *XRCC1 *gene in squamous cell carcinoma of the head and neck (HNSCC) patients and the controls.

**Genotype Allele**	**HNSCC patients** **(n = 92)** **Number (frequency)**	**Controls** **(n = 124)** **Number (frequency)**	**OR (95% CI)**
Arg/Arg	71 (0.86)	102 (0.82)	1 (reference)
Arg/Trp	21 (0.14)	22 (0.18)	1.37 (0.70; 2.68)
Trp/Trp	0 (0.00)	0 (0.00)	---------
Arg	163 (0.98)	226 (0.91)	1 (reference)
Trp	21 (0.12)	22 (0.09)	1.32 (0.70; 2.49)

**Table 3:** Distribution of genotypes and frequency of alleles of the Arg/Gln 399 (G/A 28152 exon 9) polymorphism of *XRCC1 *gene in squamous cell carcinoma of the head and neck (HNSCC) patients and the controls.

**Genotype Allele**	**HNSCC patients** **(n = 92)** **Number (frequency)**	**Controls** **(n = 124)** **Number (frequency)**	**OR (95% CI)**
Arg/Arg	37 (0,40)	49 (0.40)	1 (reference)
Arg/Gln	44 (0.48)	53 (0.43)	1.10 (0.61; 1.97)
Gln/Gln	11 (0.12)	22 (0.18)	0.66 (0.29; 1.53)
Arg	118 (0.64)	151 (0.61)	1 (reference)
Gln	66 (0.36)	97 (0.39)	0.87 (0.59; 1.29)

**Table 4:** Haplotypes distribution and frequencies of *XRCC1 *gene polymorphisms in squamous cell carcinoma of the head and neck (HNSCC) patients and the controls.

**Haplotypes XRCC1-194–399**	**HNSCC patients** **(n = 92)** **Number (frequency)**	**Controls** **(n = 124)** **Number (frequency)**	**OR (95% CI)**
Arg/Arg-Arg/Arg	29 (0,32)	43 (0,35)	1 (reference)
Arg/Trp-Arg/Arg	12 (0.13)	6 (0.05)	2.96 (1.01; 8.80)
Trp/Trp-Arg/Arg	0 (0.00)	0 (0.00)	---------
Arg/Arg-Arg/Gln	36 (0.39)	40 (0.32)	1.33 (0.70; 2.56)
Arg/Trp-Arg/Gln	8 (0,09)	13 (0,10)	0.91 (0.34; 2.48)
Trp/Trp-Arg/Gln	0 (0.00)	0 (0.00)	---------
Arg/Arg-Gln/Gln	6 (0.07)	19 (0.15)	0.47 (0.17; 1.31)
Arg/Trp-Gln/Gln	1 (0.01)	3 (0.02)	0.49 (0.05; 4.99)
Trp/Trp-Gln/Gln	0 (0,00)	0 (0,00)	---------

We also analyzed the distribution of genotypes and frequency of alleles in groups of patients suffer head and neck cancer according to different cancer staging by TNM classification (table 5 and table 6). We did not find any association of the Arg194Tyr or Arg399Gln polymorphisms in patients group with cancer progression assessed by with tumour size (T) and node status (N). Additionally, as a high risk factor for head and neck cancer occurrence we analysed patients with positive smoking status within HNSCC group according to smokers selected from controls (table 7 and table 8). While, no statistically significant differences in distribution of the Arg194Tyr genotype was calculated, we found statistically significant associations of Arg399Gln polymorphic variants of XRCC1 gene with cancer risk within smoking group of HNSCC patients. We found that Arg399Gln genotype frequency (OR, 2.70; 95% CI, 1.26–5.78) and Gln^399 ^allele (OR, 4.31; 95% CI, 2.29–8.13) was associated with patients group smoked ten or more cigarettes per day for at least ten years. On the other hand Arg399Arg wild-type genotype (OR, 0.18; 95% CI, 0.08–0.39) and Arg^399 ^allele (OR, 0.22; 95% CI, 0.12–0.41) had protective effect on cancer risk even in patients group with positive smoking status.

**Table 5:** The genotype and allele frequency and odds ratios (OR) of the Arg194Trp polymorphism of *XRCC1 *gene in patients with head and neck cancer with different tumor size and lymph node status.

	**Tumour size (T)**			**Node status (N)**		
	
**Genotype Allele**	T3 + T4 Number/Frequency	T1+ T2 Number/Frequency	OR (95% CI)	N1 + N2 + N3 Number/Frequency	N0 Number/Frequency	OR (95% CI)
Arg/Arg	43 (0.72)	28 (0.88)	0.36 (0.11 – 1.19)	20 (0.67)	51 (0.82)	0.43 (0.16 – 1.67)
Arg/Trp	17 (0.28)	4 (0.12)	2.76 (0.84 – 9.08)	10 (0.33)	11 (0.18)	2.32 (0.85 – 6.30)
Trp/Trp	0 (0.00)	0 (0.00)	---------	0 (0.00)	0 (0.00)	---------
Arg	103 (0.86)	60 (0.96)	0.40 (0.13 – 1.27)	50 (0.83)	113 (0.91)	0.48 (0.19 – 1.32)
Trp	17 (0.14)	4 (0.14)	2.47 (0.80 – 7.70)	10 (0.17)	11 (0.09)	2.05 (0.82 – 5.14)

**Table 6:** The genotype and allele frequency and odds ratios (OR) of the Arg399Gln polymorphism of *XRCC1 *gene in patients with head and neck cancer with different tumor size and lymph node status.

	**Tumour size (T)**			**Node status (N)**		
	
**Genotype Allele**	T3 + T4 Number/Frequency	T1+ T2 Number/Frequency	OR (95% CI)	N1 + N2 + N3 Number/Frequency	N0 Number/Frequency	OR (95% CI)
Arg/Arg	24 (0.40)	13 (0.41)	0,97 (0.41 – 2.34)	8 (0,27)	29 (0.47)	0.41 (0.16 – 1.07)
Arg/Gln	30 (0.50)	14 (0.44)	1.28 (0.54 – 3.05)	17 (0.57)	27 (0.44)	1.70 (0.70 – 4.08)
Gln/Gln	6 (0.10)	5 (0.16)	0.60 (0.17 – 2.14)	5 (0.17)	6 (0.10)	1.86 (0.52 – 6.70)
Arg	78 (0.65)	40 (0.62)	1.11 (0.59 – 2.09)	33 (0.55)	85 (0.69)	0.56 (0.30 – 1.06)
Gln	42 (0.35)	24 (0.38)	0.89 (0.48 – 1.68)	27 (0.45)	39 (0.31)	1.78 (0.94 – 3.36)

**Table 7:** The genotype and allele frequency and odds ratios (OR) of the Arg194Trp polymorphism of *XRCC1 *gene in squamous cell carcinoma of the head and neck (HNSCC) patients and the controls with positive smoking status.

**Genotype Allele**	**HNSCC patients** **(n = 66)** **Number (frequency)**	**Controls** **(n = 52)** **Number (frequency)**	OR (95% CI)
Arg/Arg	49 (0.74)	44 (0.85)	0.52 (0.20 – 1.33)
Arg/Trp	17 (0.26)	8 (0.15)	1.91 (0.75 – 4.85)
Trp/Trp	0 (0.00)	0 (0.00)	---------
Arg	115 (0.87)	96 (0.92)	0.56 (0.23 – 1.36)
Trp	17 (0.13)	8 (0.08)	1.77 (0.73 – 4.28)

**Table 8:** The genotype and allele frequency and odds ratios (OR) of the Arg399Gln polymorphism of *XRCC1 *gene in squamous cell carcinoma of the head and neck (HNSCC) patients and the controls with positive smoking status.

**Genotype Allele**	**HNSCC patients** **(n = 66)** **Number (frequency)**	**Controls** **(n = 52)** **Number (frequency)**	OR (95% CI)
Arg/Arg	19 (0.29)	36 (0.69)	0.18 (0.08 – 0.39)
Arg/Gln	36 (0.55)	16 (0.31)	2.70 (1.26 – 5.78)
Gln/Gln	11 (0.16)	0 (0.00)	---------
Arg	74 (0.56)	88 (0.85)	0.22 (0.12 – 0.41)
Gln	58 (0.44)	16 (0.15)	4.31 (2.29 – 8.13)

The *XRCC1 *gene polymorphisms have been extensively studied in the association with various human cancers mostly breast, lung or head and neck carcinomas. Major studies of head and neck cancer has been focused on polymorphisms of genes encoding enzymes of xenobiotic metabolism and DNA repair [32,40]. Carriers of the *XRCC1 *Arg399Gln variant have been shown to have higher levels of DNA adducts [16] and to be at greater risk for ionizing radiation sensitivity [17] and tobacco correlated DNA damage [18-20]. Recently, Sreeja at al (2008) has shown that the carriers of *XRCC1 *Gln399Gln genotypes were at higher risk of lung cancer [12]. On the other hand, López-Cima et al. (2007) has been reported that individuals homozygous for the *XRCC1 *Gln^339 ^allele presented no risk of developing lung cancer [6]. The association between *XRCC1 *Arg399Gln polymorphism and ductal carcinoma of women with breast cancer was found statistically significant in studies performed by Dufloth et al. at 2008 [13]. Despite of large number of studies, in well-characterized populations, results from HNSCC patients are still confusing. There was a marginally significant risk of HNSCC observed in variants of *XRCC1 *genotype with Trp^194 ^allele in Thailand population [41]. No altered risk was associated with the *XRCC1 *Arg399Gln genotype in Li et al. studies [42], however smokers carrying risk genotype of *XRCC1 *with dominant Gln^399 ^allele were over-represented in head and neck cancer populations from eastern region of India [43]. Recently, combinational polymorphisms of four DNA repair genes *XRCC1, XRCC2, XRCC3*, and *XRCC4 *and their association with HNSCC cancer in Taiwan has been investigated. [14]. Except for *XRCC2*, none of SNPs was found to individually contribute to cancer risk. In our study, we found that Gln^399 ^allele may also increase head and neck cancer risk in population with positive smoking status. Finally, no association was found individually for either analyzed SNPs but we evidenced that combined genotypes of *XRCC1 *may have impact on HNSCC risk.

## Conclusion

Head and neck cancer patients have variable prognoses even within the same clinical stage and while receiving similar treatments. The number of studies of genetic polymorphisms as prognostic factors of HNSCC outcomes is growing. Candidate polymorphisms have been evaluated in DNA repair, cell cycle, xenobiotic metabolism, and growth factor pathways. In our study, we assessed two common polymorphisms of the *XRCC1 *gene that might influence DNA repair capacity and their association with head and neck cancer risk. Finally, we identified the combined genotype of Arg194Trp-Arg399Arg that was associated with HNSCC cancer risk and may have an impact on identification of a high-risk population.

## Competing interests

The authors declare that they have no competing interests.

## Authors' contributions

MK have made substantial contributions to conception, design and drafting the manuscript. KP, PR, WP, AB-K and JS have made acquisition of data, analysis and interpretation of data. WM, JO, AM-S have made substantial contributions to patients sample collection. IM has made substantial contributions to conception and design, acquisition of data, analysis and interpretation of data, drafting the manuscript and revising it critically for important intellectual content. He has also given final approval of the version to be published.

## References

[B1] Lindahl T, Wood RD (1999). Quality control by DNA repair. Science.

[B2] Hoeijmakers JH (2001). Genome maintenance mechanisms for preventing cancer. Nature.

[B3] Barnes DE, Lindahl T (2004). Repair and genetic consequences of endogenous DNA base damage in mammalian cells. Annu Rev Genet.

[B4] Vogelstein B, Kinzler KW (2004). Cancer genes and the pathways they control. Nat Med.

[B5] Mohrenweiser HW, Wilson DM, Jones IM (2003). Challenges and complexities in estimating both the functional impact and the disease risk associated with the extensive genetic variation in human DNA repair genes. Mutat Res.

[B6] López-Cima MF, González-Arriaga P, García-Castro L, Pascual T, Marrón MG, Puente XS, Tardón A (2007). Polymorphisms in XPC, XPD, XRCC1, and XRCC3 DNA repair genes and lung cancer risk in a population of northern Spain. BMC Cancer.

[B7] Martinez-Balibrea E, Manzano JL, Martinez-Cardus A, Moran T, Cirauqui B, Catot S, Taron M, Abad A (2007). Combined analysis of genetic polymorphisms in thymidylate synthase, uridine diphosphate glucoronosyltransferase and X-ray cross complementing factor 1 genes as a prognostic factor in advanced colorectal cancer patients treated with 5-fluorouracil plus oxaliplatin or irinotecan. Oncol Rep.

[B8] Burri RJ, Stock RG, Cesaretti JA, Atencio DP, Peters S, Peters CA, Fan G, Stone NN, Ostrer H, Rosenstein BS (2008). Association of single nucleotide polymorphisms in SOD2, XRCC1 and XRCC3 with susceptibility for the development of adverse effects resulting from radiotherapy for prostate cancer. Radiat Res.

[B9] McWilliams RR, Bamlet WR, Cunningham JM, Goode EL, de Andrade M, Boardman LA, Petersen GM (2008). Polymorphisms in DNA repair genes, smoking, and pancreatic adenocarcinoma risk. Cancer Res.

[B10] Fontana L, Bosviel R, Delort L, Guy L, Chalabi N, Kwiatkowski F, Satih S, Rabiau N, Boiteux JP, Chamoux A, Bignon YJ, Bernard-Gallon DJ (2008). DNA repair gene ERCC2, XPC, XRCC1, XRCC3 polymorphisms and associations with bladder cancer risk in a French cohort. Anticancer Res.

[B11] Wang Z, Xu B, Lin D, Tan W, Leaw S, Hong X, Hu X (2008). XRCC1 polymorphisms and severe toxicity in lung cancer patients treated with cisplatin-based chemotherapy in Chinese population. Lung Cancer.

[B12] Sreeja L, Syamala VS, Syamala V, Hariharan S, Raveendran PB, Vijayalekshmi RV, Madhavan J, Ankathil R (2008). Prognostic importance of DNA repair gene polymorphisms of XRCC1 Arg399Gln and XPD Lys751Gln in lung cancer patients from India. J Cancer Res Clin Oncol.

[B13] Dufloth RM, Arruda A, Heinrich JK, Schmitt F, Zeferino LC (2008). The investigation of DNA repair polymorphisms with histopathological characteristics and hormone receptors in a group of Brazilian women with breast cancer. Genet Mol Res.

[B14] Yen CY, Liu SY, Chen CH, Tseng HF, Chuang LY, Yang CH, Lin YC, Wen CH, Chiang WF, Ho CH, Chen HC, Wang ST, Lin CW, Chang HW (2008). Combinational polymorphisms of four DNA repair genes XRCC1, XRCC2, XRCC3, and XRCC4 and their association with oral cancer in Taiwan. J Oral Pathol Med.

[B15] Shall S, de Murcia G (2000). Poly(ADP-ribose) polymerase-1: what have we learned from the deficient mouse model?. Mutat Res.

[B16] Lunn RM, Langlois RG, Hsieh LL, Thompson CL, Bell DA (1999). *XRCC1 *polymorphisms: effects on aflatoxin B1-DNA adducts and glycophorin A variant frequency. Cancer Res.

[B17] Hu JJ, Smith TR, Miller MS (2001). Amino acid substitution variants of APE1 and *XRCC1 *genes associated with ionizing radiation sensitivity. Carcinogenesis.

[B18] Duell EJ, Wiencke JK, Cheng TJ, Varkonyi A, Zuo ZF, Ashok TD, Mark EJ, Wain JC, Christiani DC, Kelsey KT (2000). Polymorphisms in the DNA repair genes *XRCC1 *and ERCC2 and biomarkers of DNA damage in human blood mononuclear cells. Carcinogenesis.

[B19] Abdel-Rahman SZ, El Zein RA (2000). The 399Gln polymorphism in the DNA repair gene *XRCC1 *modulates the genotoxic response induced in human lymphocytes by the tobacco-specific nitrosamine NNK. Cancer Lett.

[B20] Lei YC, Hwang SJ, Chang CC, Kuo HW, Luo JC, Chang MJ, Cheng TJ (2002). Effects on sister chromatid exchange frequency of polymorphisms in DNA repair gene *XRCC1 *in smokers. Mutat Res.

[B21] Kubota Y, Nash RA, Klungland A, Schar P, Barnes DE, Lindahl T (1996). Reconstitution of DNA base excision-repair with purified human proteins: interaction between DNA polymerase beta and the *XRCC1 *protein. EMBO J.

[B22] Caldecott KW (2003). *XRCC1 *and DNA strand break repair. DNA Repair.

[B23] Marsin S, Vidal AE, Sossou M, Ménissier-de Murcia J, Le Page F, Boiteux S, de Murcia G, Radicella JP (2003). Role of XRCC1 in the coordination and stimulation of oxidative DNA damage repair initiated by the DNA glycosylase hOGG1. J Biol Chem.

[B24] Campalans A, Marsin S, Nakabeppu Y, O'connor TR, Boiteux S, Radicella JP (2005). XRCC1 interactions with multiple DNA glycosylases: a model for its recruitment to base excision repair. DNA Repair (Amst).

[B25] Nazarkina ZK, Khodyreva SN, Marsin S, Lavrik OI, Radicella JP (2007). XRCC1 interactions with base excision repair DNA intermediates. DNA Repair (Amst).

[B26] Koskinen WJ (2006). Prognostic markers in head and neck carcinoma. Academic Dissertation.

[B27] Zhou W, Liu G, Miller DP, Thurston SW, Li Lian X, Wain JC, Lynch TJ, Li S, Christiani DC (2002). Gene-environment interaction for the ERCC2 polymorphisms and cumulative cigarette smoking exposure in lung cancer. Cancer Res.

[B28] Stern MC, Siegmund KD, Corral R, Haile RW (2005). XRCC1 and XRCC3 Polymorphisms and Their Role as Effect Modifiers of Unsaturated Fatty Acids and Antioxidant Intake on Colorectal Adenomas Risk. Cancer Epidemiol Biomarkers Prev.

[B29] Helzlsouer KJ, Harris EL, Parshad R, Perry HR, Price FM, Sanford KK (1996). DNA repair proficiency: potential susceptiblity factor for breast cancer. J Natl Cancer Inst.

[B30] Wei Q, Spitz MR (1997). The role of DNA repair capacity in susceptibility to lung cancer: a review. Cancer Metastasis Rev.

[B31] Hopkins J, Cescon DW, Tse D, Bradbury P, Xu W, Ma C, Wheatley-Price P, Waldron J, Goldstein D, Meyer F, Bairati I, Liu G (2008). Genetic polymorphisms and head and neck cancer outcomes: a review. Cancer Epidemiol Biomarkers Prev.

[B32] Hiyama T, Yoshihara M, Tanaka S, Chayama K (2008). Genetic polymorphisms and head and neck cancer risk (Review). Int J Oncol.

[B33] Lindahl T (2001). Keynote: past, present, and future aspects of base excision repair. Prog Nucleic Acid Res Mol Biol.

[B34] Hoeijmakers JH (2001). Genome maintenance mechanisms for preventing cancer. Nature.

[B35] Bohr VA (2002). DNA damage and its processing: relation to human disease. J Inherit Metab Dis.

[B36] Mohrenweiser HW, Wilson DM, Jones IM (2003). Challenges and complexities in estimating both the functional impact and the disease risk associated with the extensive genetic variation in human DNA repair genes. Mutat Res.

[B37] Monaco R, Rosal R, Dolan MA, Pincus MR, Brandt-Rauf PW (2007). Conformational effects of a common codon 399 polymorphism on the BRCT1 domain of the XRCC1 protein. Protein J.

[B38] Olshan AF, Watson MA, Weissler MC, Bell DA (2002). XRCC1 polymorphisms and head and neck cancer. Cancer Lett.

[B39] Nelson HH, Kelsey KT, Mott LA, Karagas MR (2002). The XRCC1 Arg399Gln polymorphism, sunburn, and non-melanoma skin cancer: evidence of gene-environment interaction. Cancer Res.

[B40] Harth V, Schafer M, Abel J, Maintz L, Neuhaus T, Besuden M, Primke R, Wilkesmann A, Thier R, Vetter H, Ko YD, Bruning T, Bolt HM, Ickstadt K (2008). Head and neck squamous-cell cancer and its association with polymorphic enzymes of xenobiotic metabolism and repair. J Toxicol Environ Health A.

[B41] Kietthubthew S, Sriplung H, Au WW, Ishida T (2006). Polymorphism in DNA repair genes and oral squamous cell carcinoma in Thailand. Int J Hyg Environ Health.

[B42] Li C, Hu Z, Lu J, Liu Z, Wang LE, El-Naggar AK, Sturgis EM, Spitz MR, Wei Q (2007). Genetic polymorphisms in DNA base-excision repair genes ADPRT, XRCC1, and APE1 and the risk of squamous cell carcinoma of the head and neck. Cancer.

[B43] Majumder M, Sikdar N, Paul RR, Roy B (2005). Increased risk of oral leukoplakia and cancer among mixed tobacco users carrying XRCC1 variant haplotypes and cancer among smokers carrying two risk genotypes: one on each of two loci, GSTM3 and XRCC1 (Codon 280). Cancer Epidemiol Biomarkers Prev.

